# Intracranial Myeloid Sarcoma as the First Manifestation of Acute Monoblastic Leukemia

**DOI:** 10.1055/a-2914-7953

**Published:** 2026-07-27

**Authors:** Bernardo Ramos Fraga, Juan Francisco Tovar Gaucin, Vicente Jonathan Sánchez Coronado, Pablo Josafat Avalos Montes, Silvia Judith Hernández Martínez, José Ramón Olivas Campos

**Affiliations:** 1 Department of Neurosurgery, UMAE Hospital de Especialidades No. 25, Centro Medico Nacional Noreste, Instituto Mexicano del Seguro SocialMonterreyNuevo LeonMéxico; 2 Department of NeuropathologyUMAE Hospital de Especialidades No. 25, Centro Médico Nacional Noreste, Instituto Mexicano del Seguro SocialMonterreyNuevo LeónMéxico

**Keywords:** intracranial myeloid sarcoma, orbitozygomatic craniotomy, acute monoblastic leukemia, extramedullary myeloid tumor

## Abstract

**Background** Myeloid sarcoma, also called granulocytic sarcoma, extramedullary myeloid tumor, or chloroma, is a manifestation of a myeloid neoplasm. These tumors can develop in various anatomical sites, including the central nervous system, presenting as an extra-axial tumor affecting the meninges or the ependyma. Diagnosis is challenging due to its resemblance to other intracranial tumors such as meningiomas.

**Case Description** We present the case of a 17-year-old male with intermittent left frontotemporal headache, diplopia during horizontal gaze, and proptosis of the left eye. During the imaging evaluation, an extra-axial tumor was observed, suggesting a meningothelial lesion of the left sphenoid wing. The patient had no previous hematological disease. Tumor resection was performed using an orbitozygomatic approach. After 2 months, the patient had recurrence of the lesion associated with spontaneous epistaxis and leucocytosis and thrombocytopenia. Bone marrow aspirate biopsy and histopathology results were consistent with myeloid sarcoma.

**Conclusion** Intracranial myeloid sarcomas mimic meningiomas, metastases, lymphomas, and even Ewing’s sarcoma, making it difficult to suspect myeloid sarcoma in a patient without prior hematologic disease. Surgery aids in diagnosis through biopsy or reduces the volume effect that could cause neurological deficit.

## Introduction

Myeloid neoplasms can manifest as tumors or extramedullary masses, termed myeloid sarcomas. These are also known as granulocytic sarcomas, extramedullary myeloid tumors, or chloromas (named for their greenish hue caused by the enzyme myeloperoxidase). 1 2 Myeloid sarcomas (MS) may precede acute myeloid leukemia (AML), coexist with it, or emerge as a blastic transformation in chronic myeloproliferative disorders or myelodysplastic syndromes. They may also indicate a relapse in previously treated cases.

These tumors can develop in various anatomical locations, including lymphoid organs, bones (such as the skull and orbit), skin, soft tissues, mucosal surfaces, and internal organs. In rare instances (5–14% of cases), they affect the central nervous system (CNS). 3 When involving the CNS, they typically present as extra-axial lesions affecting the meninges or ependyma. However, diagnosis is often challenging due to their resemblance to other intracranial tumors like meningiomas, metastases, Ewing’s sarcomas, and lymphomas. 4

We present a rare case of intracranial myeloid sarcoma (IMS) in a patient with no prior hematological disorder. Clinical presentation, differential diagnoses, and subsequent progression post-treatment are discussed.

## Case Presentation

A 17-year-old male presented with a 15-day history of intermittent left frontotemporal headache, diplopia during leftward gaze, and proptosis of the left eye. No other significant symptoms were noted. Physical examination revealed diminished visual acuity and restricted adduction of the left eye but no murmur or pulsation ( Fig. 1 ). Laboratory findings at admission were unremarkable ( Table 1 ). Computed tomography (CT) imaging identified a hyperdense lesion in the left middle fossa originating from the sphenoid wing and associated with adjacent bone density reduction. Magnetic resonance imaging (MRI) revealed an iso- to hypointense lesion on T1- and T2-weighted images relative to gray matter with homogeneous contrast enhancement ( Fig. 2 ). The lesion compressed the temporal pole and invaded the lateral wall of the ipsilateral orbit and infratemporal fossa. With these imaging findings, a tumor of meningothelial origin was suspected, so a family history of intracranial or spinal tumors was asked to rule out a hereditary syndrome; however, these were denied and an MRI of the spine was unremarkable.

**Fig. 1:**
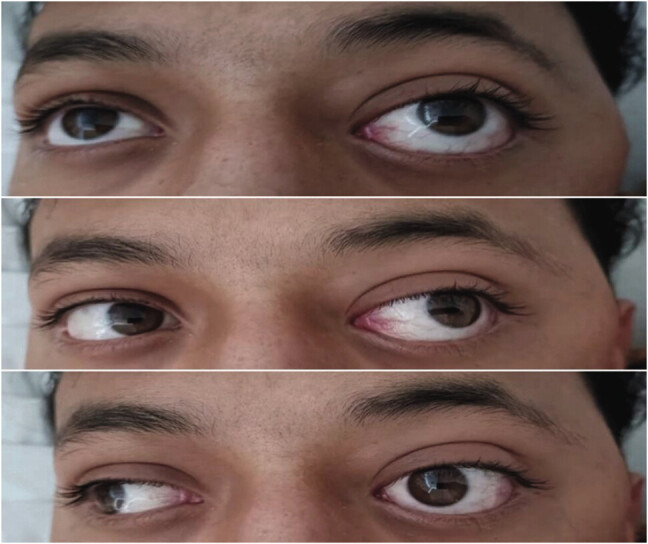
Limited eye movements are observed due to adduction loss of the left eye.

**Table 1:** Laboratory findings on admission and readmission.

Parameter	Values at admission	Values at readmission
WBC (K/μL)	5.316	22.740
Platelets (× $10^{9}$ /L)	193.0	98.7
Neutrophil (K/μL)	2.5	2.0
Lymphocyte (K/μL)	1.7	2.0
Monocyte (K/μL)	0.5	17.5
Basophil (K/μL)	0.0	1.0

**Fig. 2:**
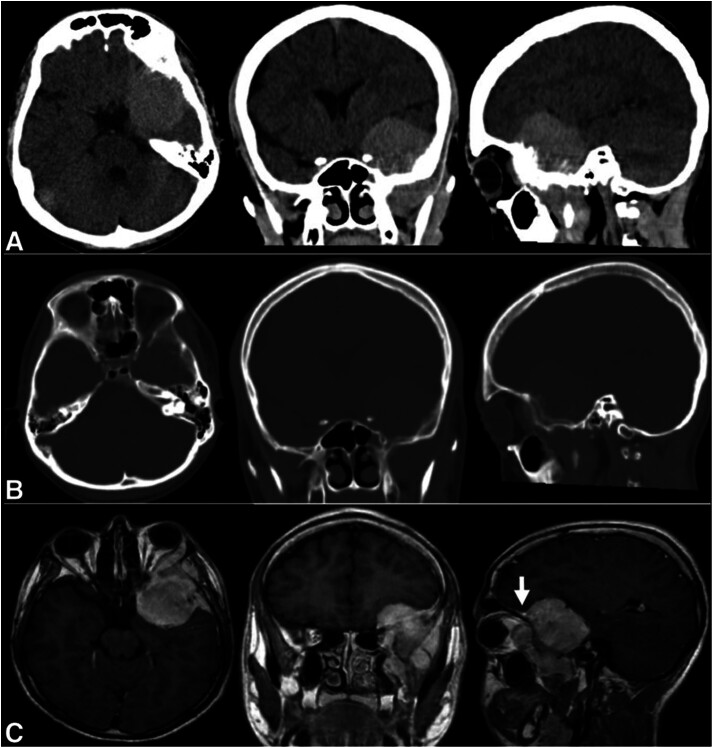
( **A** , **B** ) CT imaging of the skull showing a hyperdense lesion with decreased adjacent bone density. ( **C** ) MRI showing contrast enhancement of the lesion, compression of the temporal pole, invasion of the lateral wall of the orbit, and its extension into the infratemporal fossa. Note the similarity to a dural tail (arrow) in the anterior skull base.

Suspecting a sphenoid wing meningioma, surgical resection was performed using a left orbitozygomatic craniotomy. During surgery, as the skin flap dissection was being performed, increased bleeding and friability in the temporal muscle, and adjacent porous bone were noted ( Fig. 3 ). Debulking of the lesion was performed and the sample obtained was sent to pathology for further analysis. Postoperative CT revealed its complete removal ( Fig. 4 ). The patient showed clinical improvement with restored ocular movements and reduced proptosis and was then discharged. However, he was lost medical follow-up, so no information was obtained about his postsurgical condition. After 2 months, he returned with a 2-day history of oppressive frontoparietal headache, horizontal diplopia during leftward gaze, and spontaneous epistaxis on three occasions. Laboratory tests revealed leukocytosis with elevated monocytes, low neutrophils, and moderate thrombocytopenia ( Table 1 ). CT imaging showed recurrence of the lesion in the left middle fossa infiltrating the orbit and infratemporal fossa ( Fig. 5 ).

**Fig. 3:**
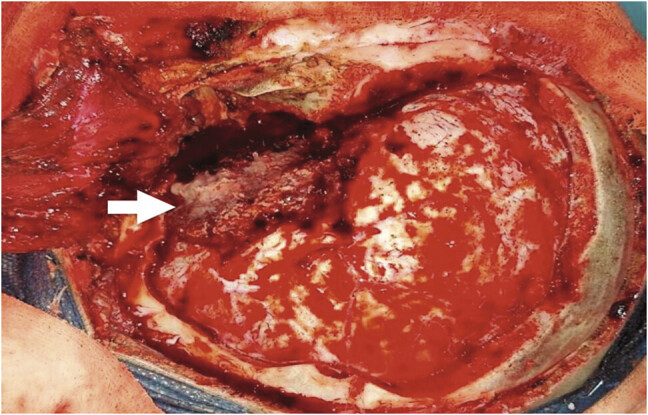
Intraoperative image of orbitozygomatic craniotomy. The epidural lesion can be observed during the dural exposure (arrow).

**Fig. 4:**
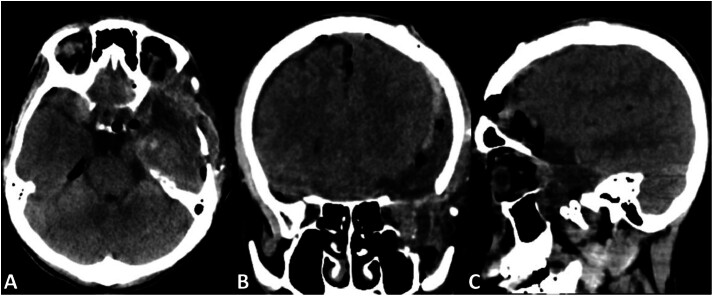
Postoperative axial ( **A** ), coronal ( **B** ), and sagittal ( **C** ) CT imaging showing complete resection of the lesion.

**Fig. 5:**
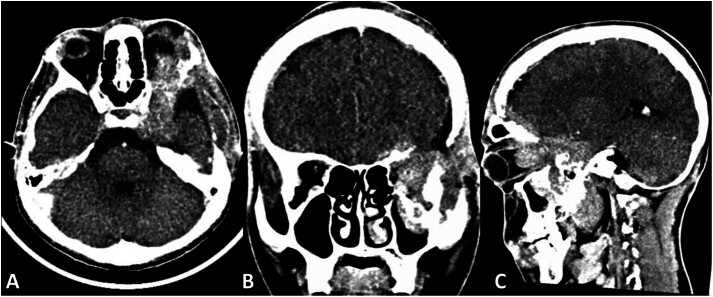
Axial ( **A** ), coronal ( **B** ), and sagittal ( **C** ) CT imaging showing recurrence of the lesion in the temporal and infratemporal fossa with invasion of the left orbit.

Based on imaging and laboratory findings, myeloid sarcoma was suspected. Initial histopathological analysis of samples from the lesion, muscle, and temporal bone was consistent with IMS infiltration ( Figs. 6 and 7 ). A bone marrow aspiration (BMA) ( Fig. 8 ) was performed by the Hematology Department and an analysis with immunohistochemical markers of the lesion samples showed positivity for CD45, CD33, and CD13 ( Fig. 9 ) and negative markers for CD3, CD20, CD38, CD56, CD138, EMA, TdT, and Cyclin-D1. Therefore, a diagnosis of acute monoblastic leukemia was made. The patient was then submitted for chemotherapy treatment that led to remission, but declined allogeneic hematopoietic stem cell transplantation. He regained conjugated eye movements and the proptosis of the left eye improved ( Fig. 10 ).

**Fig. 6:**
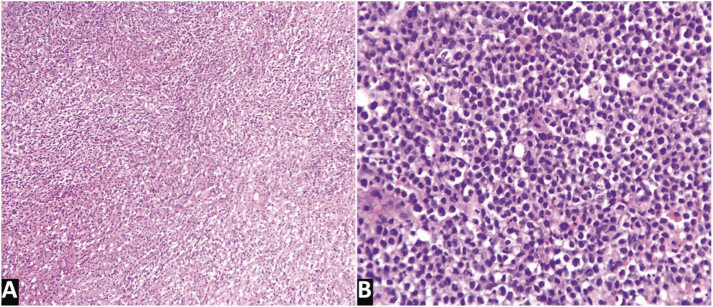
( **A, B** ) Histopathological examination showing proliferation of malignant cells with scant cytoplasm, large, hyperchromatic, round to oval nuclei with clefts. H&E staining. **A** (10×), **B** (40×).

**Fig. 7:**
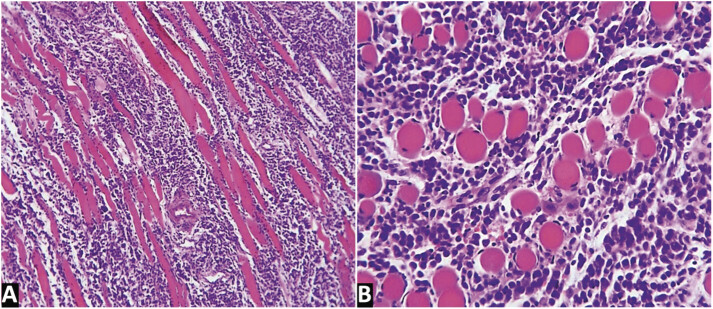
( **A, B** ) Histopathological examination showing infiltration of striated muscle by neoplastic cells with scant cytoplasm, cleft nuclei, and some discrete nucleoli corresponding to blasts. H&E staining. **A** (10×), **B** (40×).

**Fig. 8:**
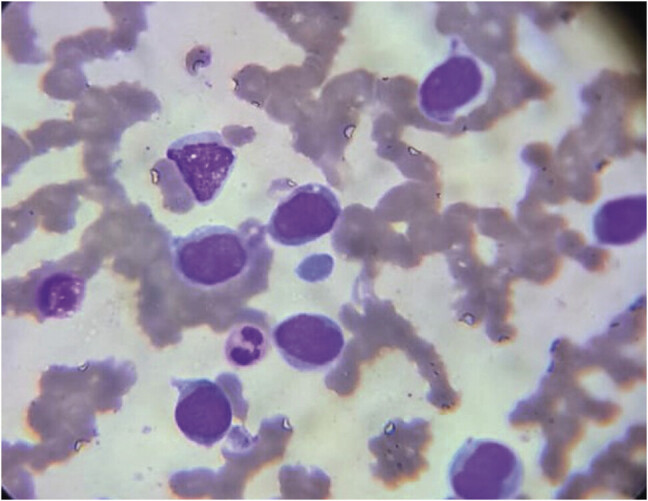
Bone marrow aspiration showing large blasts and bilobed nuclei. Wright staining (100×).

**Fig. 9:**
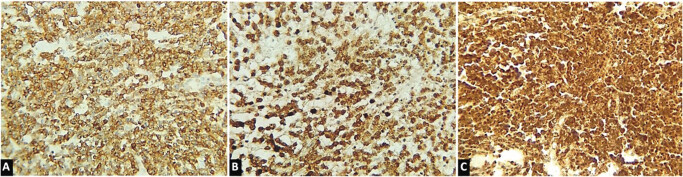
Neoplastic cells showing positivity for immunohistochemical markers CD45 ( **A** ), CD33 ( **B** ), and CD13 ( **C** ).

**Fig. 10:**
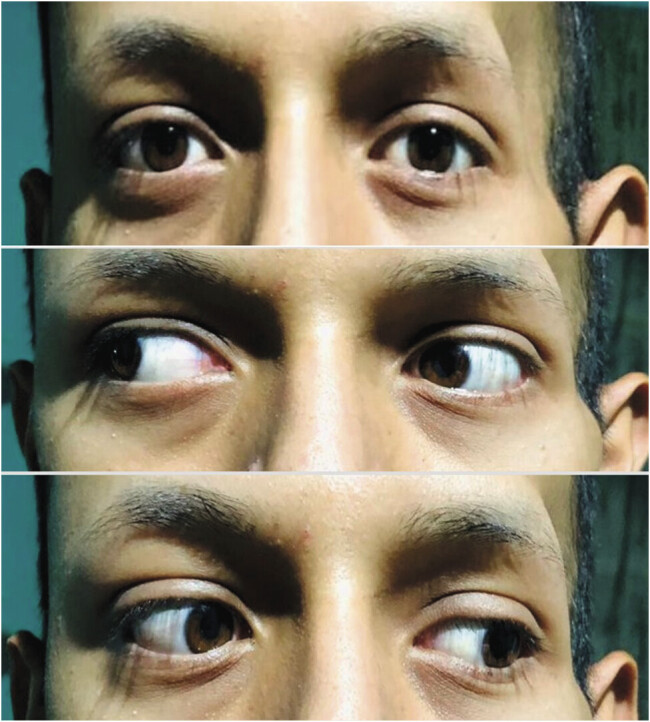
Recovery of eye movements with a central primary position, and adequate right and left lateralization.

## Discussion

IMS is an uncommon manifestation of AML. Four developmental patterns have been identified: (a) primary IMS preceding AML (1–25% of cases), (b) IMS concurrent with AML diagnosis (15–35%), (c) isolated IMS recurrence without medullary relapse within 30 days, and (d) IMS accompanying AML relapse in bone marrow. 5 Pathophysiological theories suggest aberrant localization signals for leukemic blasts that precludes the more common bone marrow localization. This may represent a subclone of an original AML clone in cases of concurrent or knockdown presentation. 2 IMS may result from hematogenous dissemination of leukemic cells adhering to vessel walls and proliferating intracranially. Approximately 54% of IMS cases are intra-axial while 45% are extra-axial. 6 7

### Diagnostic Approach

Due to its rarity and lack of preexisting hematological disorders or signs of systemic disease in some patients, IMS is often misdiagnosed based on clinical presentation, radiological findings, and even histopathological results. 8 Misdiagnoses, the diffuse large B cell lymphoma being the most common, are seen in approximately 25% of cases where IMS precedes AML diagnosis by months or even years. 9 10

In this case study, due to the absence of any hematological preexistent diagnosis, initial suspicion was a meningioma, based on typical imaging characteristics for such lesions—hyperdense appearance on CT scans and iso- to hypointense signals on T1- and T2-weighted MRI scans with homogeneous enhancement, 11 and was addressed with its standard management. However, it has been suggested that advanced imaging techniques like perfusion MRI may help differentiate IMS from other similar lesions. 12

Preoperative diagnosis remains challenging; thus, cytological analysis of cerebrospinal fluid alongside chromosomal studies and molecular techniques are vital for accurate identification. Definitive diagnosis of an MS relies on pathological evaluation supported by immunohistochemical staining using monoclonal antibodies against myeloperoxidase. 13 14

### Management Approach

Evidence regarding surgical intervention’s impact on mortality or recurrence rates in IMS is limited. A meta-analysis by Lee et al, encompassing 99 patients from which 51 underwent surgical resection for IMS, reported total macroscopic resection in only 24.3% of cases while subtotal resection or biopsy was performed in 75.7%. Most common localizations were: the temporal lobe (10.3%), the cerebellum (10.3%), and the falcine/parasagittal region (10.3%) of the brain. Mortality occurred in 41.8% and recurrence in 43.8%, and surgical complications associated with tumoral bleeding, which ended in consequent transtentorial herniation, and neurological sequelae arose in 11.5% during follow-up. 15 Therefore, they concluded that surgery, extent of resection, and timing of IMS diagnosis relative to AML diagnosis were not significantly associated with mortality.

Surgery primarily aids in diagnosis via biopsy or alleviates mass effects causing neurological deficits, where decompression of neural structures and surgical cytoreduction improve patients’ functional status and, consequently, their quality of life. However, the extent of surgical resection does not appear to be associated with improved survival and may not be justified in most cases. In this case study, surgery improved functional status by resolving ophthalmoplegia and reducing diplopia but did not influence survival outcomes directly.

Optimal MS treatment strategy is complex and requires taking into account patient-specific factors (age, comorbidities, personal wishes), disease biology (karyotype/mutations), donor availability for hematopoietic stem cell transplantation (HSCT), anatomical tumor location/size, disease status (isolated vs. synchronous MS, presence of de novo/relapsed/refractory disease), and even the relapse risk post-HSCT. 16 Chemotherapy combined with radiotherapy remains central to improving survival outcomes.

This case report is noteworthy not only for its rarity but also for its unique presentation as an IMS without prior hematological disease, posing a significant diagnostic challenge based solely on imaging. In patients with atypical intraoperative findings and a preoperative suspicion of meningothelial tumor, it is crucial to include this entity in the differential diagnosis, as early identification significantly influences prognosis.

## Conclusion

IMS can mimic other more common tumors in the CNS, such as meningiomas, metastases, lymphomas, and even Ewing’s sarcomas, making it difficult to suspect in a patient without prior hematologic disease. Chemotherapy, radiotherapy, and HSCT are the recommended treatments for most patients. Surgery aids in diagnosis through biopsy or reduces the volume effect that could cause neurological deficit.
